# Bacterial Communities and Virulence Associated with Pine Wood Nematode *Bursaphelenchus xylophilus* from Different *Pinus* spp.

**DOI:** 10.3390/ijms20133342

**Published:** 2019-07-07

**Authors:** Qi Xue, Yang Xiang, Xiao-Qin Wu, Ming-Jie Li

**Affiliations:** 1 Co-Innovation Center for Sustainable Forestry in Southern China, College of Forestry, Nanjing Forestry University, Nanjing, Jiangsu 210037, China; 2 Jiangsu Key Laboratory for Prevention and Management of Invasive Species, Nanjing Forestry University, Nanjing, Jiangsu 210037, China

**Keywords:** *Bursaphelenchus xylophilus*, diversity, bacteria, pine, surface-sterilized, virulence

## Abstract

*Bursaphelenchus xylophilus*, the causal agent of pine wilt disease, is a destructive threat to pine forests. The role of bacteria associated with *B. xylophilus* in pine wilt disease has attracted widespread attention. This study investigated variation in bacterial communities and the virulence of surface-sterilized *B. xylophilus* from different *Pinus* spp. The predominant culturable bacteria of nematodes from different pines were *Stenotrophomonas* and *Pseudomonas*. Biolog EcoPlate analysis showed that metabolic diversity of bacteria in *B. xylophilus* from *P. massoniana* was the highest, followed by *P. thunbergii* and *P. densiflora*. High-throughput sequencing analysis indicated that bacterial diversity and community structure in nematodes from the different pine species varied, and the dominant bacteria were *Stenotrophomonas* and *Elizabethkingia*. The virulence determination of *B. xylophilus* showed that the nematodes from *P. massoniana* had the greatest virulence, followed by the nematodes from *P. thunbergii* and *P. densiflora*. After the nematodes were inoculated onto *P. thunbergii*, the relative abundance of the predominant bacteria changed greatly, and some new bacterial species emerged. Meanwhile, the virulence of all the nematode isolates increased after passage through *P. thunbergii*. These inferred that some bacteria associated with *B. xylophilus* isolated from different pine species might be helpful to adjust the PWN’s parasitic adaptability.

## 1. Introduction

The pine wilt disease (PWD) (caused by the pine wood nematode (PWN), *Bursaphelenchus xylophilus*), is one of the most destructive diseases affecting pine trees. It has resulted in huge economic losses by devastating the forest ecosystem in China, Japan and Korea [1]. This disease involves interactions among various factors including the pathogenic nematode, the vector beetle, the pine host, PWN-associated bacteria, fungi and the environment. The pathogenic mechanism of PWD is still not well understood [2,3].

Under natural conditions, *B. xylophilus* can damage 40 species of *Pinus* and nine other, non-pine species [4]. In China, *Pinus massoniana*, *Pinus thunbergii* and *Pinus densiflora* are the main hosts of PWN [5]. The nematode infects the host and migrates through cortical tissue and resin canals in pine trees [6]. Wang et al. [7] found that some bacteria carried by PWN from diseased trees were absent from healthy pines. Roriz et al. [8] inoculated *Pinus pinaster* with pine wood nematodes and found that the size and diversity of the bacterial population in the trees increased with the progress of the disease. Furthermore, bacterial species isolated from the cuticle of nematodes were similar to those isolated from the xylem of pines. Other studies showed that PWN could obtain some bacteria beneficial for itself from its natural environments [9]. The bacterial communities from nematodes collected from different *Pinus* spp. with distinct geographical locations show considerable diversity [10]. The virulence of the PWNs from different *Pinus* spp. also varied markedly [11].

Since Oku et al. [4] found that bacteria may participate in the pathogenic process of PWD, the role of bacteria associated with PWN in PWD has attracted great attention [10,12,13]. Some bacteria isolated from *B. xylophilus* can produce toxic metabolites which cause pine tree wilting [4]. Several studies have supported the observation that bacteria associated with *B. xylophilus* contributed to PWN colonization [13,14]. However, previous studies mainly focused on the bacteria present on the cuticle of PWN. The composition of the bacterial communities on the nematode cuticle varied as the PWN were exposed to features of the ambient environment, such as host plants and insect vectors. These bacteria associated with PWNs may be opportunistic [15]. Therefore, investigation into the bacteria in surface-sterilized *B. xylophilus* may help to reveal the role of bacteria in PWD. The species, carbon metabolism and gene expression of the culturable bacteria in PWNs were shown to be related to the virulence of *B. xylophilus* [16,17,18]. Xiang et al. [19] analyzed the diversity of bacteria associated with *B. xylophilus* populations with different virulence and showed that the identity of the PWN-associated bacteria might be related to the virulence of the nematodes.

Many questions remain as to whether there are any differences among the bacterial communities of the PWNs from different pine species, whether these bacterial communities change after the PWNs infect pines, and whether these changes, if any, have an influence on PWN’s parasitic adaptability. Therefore, we isolated PWNs from *P. massoniana*, *P. thunbergii* and *P. densiflora*. The diversity of culturable bacteria in surface-sterilized *B. xylophilus* from different pines was analyzed by using culture-dependent and Biolog methods. We inoculated *P. thunbergii* with PWN isolates from different pine species. The community structure of PWN-associated bacteria, both before and after inoculating *P. thunbergii*, was analyzed using high-throughput sequencing technology by amplification of the 16S rDNA region. The virulence of the PWN populations was determined and compared before and after inoculation. The results will provide useful information with which to better understand the diversity and community structure of bacteria in *B. xylophilus* from different pine species, and their relationship with PWN and the host pine.

## 2. Results

### 2.1. Culturable Bacteria in PWNs Isolated from Different Pine Species

**Figure 1:**
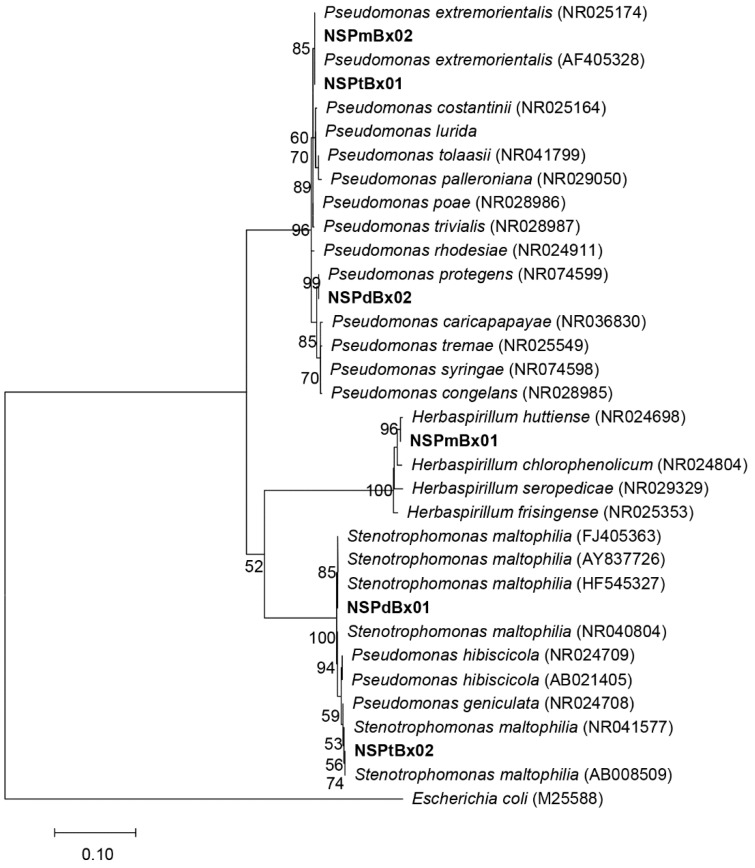
Phylogenetic trees of endo-bacteria isolated from *Bursaphelenchus xylophilus* based on 16S rDNA. Support values of bootstrap >50% are indicated at nodes.

Two bacterial isolates (labeled “NS-01” and “NS-02” from each surface-sterilized PWN isolate, e.g., NSPmBx01) were selected at random from the colonies from each PWN isolate. The phylogenetic trees of bacteria in PWNs were constructed based on 16S rDNA phylogenetic analysis (Figure 1). The resulting trees showed that the bacteria NSPmBx01 and NSPmBx02 in nematode isolate PmBx isolated from *P. massoniana* were clustered with *Herbaspirillun huttiense* and *Pseudomonas extremorientalis.* In addition, the bacterium NSPdBx01 in PdBx from *P. densiflora* showed high similarity to *Stenotrophomonas maltophilia*, whereas NSPdBx02 was clustered with *Pseudomonas protegens*. The NSPtBx01 in PtBx from *P. thunbergii* was clustered with *Pseudomonas extremorientalis*, whereas NSPtBx02 was clustered with *Stenotrophomonas maltophilia*.

### 2.2. Carbon Metabolic Activities of Bacteria in PWNs Isolated from Different Pine Species

**Figure 2:**
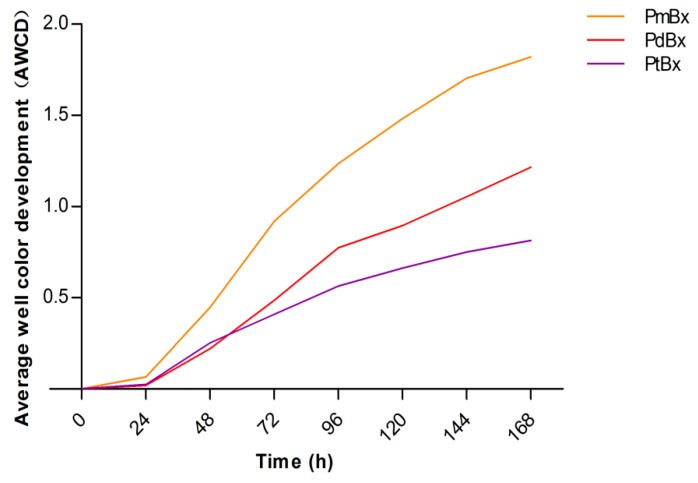
Average well color development (AWCD) values from Biolog EcoPlates for microbial community profiling of *Bursaphelenchus xylophilus* from different pine species.

Differences in carbon metabolic abilities among PWNs from the different pine species were observed. During the first 24 h after inoculation, the AWCDs of the bacteria in the PWNs were low, suggesting that the carbon sources in the wells of the Biolog EcoPlates were poorly utilized. After the first 24 h, AWCDs increased quickly. Compared with the variation in AWCD among the three samples from PWNs isolated from the three *Pinus* spp., the data showed that the bacteria in the PmBx nematodes isolated from *P. massoniana* had the highest utilization rate of carbon sources followed by the bacteria in PdBx from *P. densiflora*, with the bacteria in PtBx from *P. thunbergii* showing the lowest rate of utilization of the carbon sources (Figure 2).

### 2.3. Diversity and Evenness of Bacterial Communities in PWNs Isolated from Different Pines

Two diversity indices were calculated from the Biolog EcoPlate data to investigate the alpha diversity of the bacterial communities in *B. xylophilus* from different pine species (Table 1). The Shannon-Wiener index (*H*) of PdBx from *P. densiflora* was higher than that of PmBx from *P. massoniana*, with the *H* values of PtBx from *P. thunbergii* being the lowest, but the differences were not significant (*p* > 0.05). The Evenness index (*E*) of bacteria in PmBx was less than that in PdBx (Table 1), with the evenness of bacteria from PWN isolated from *P. densiflora* being the highest, followed by the index from *P. thunbergii* and finally that from *P. massoniana* (Table 1).

### 2.4. Variation in Carbon Source Utilization by Bacteria from PWNs Isolated from Different Pine Species

**Figure 3:**
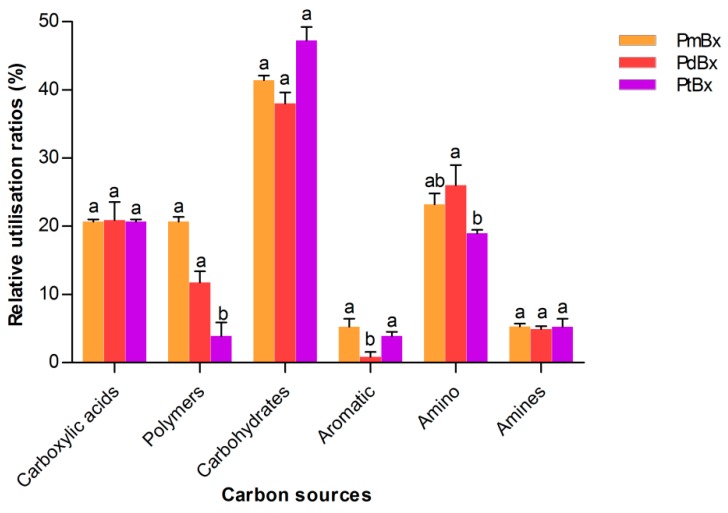
Relative utilization ratios of six groups of carbon sources by the endo-bacteria from *Bursaphelenchus xylophilus.* The bar indicates standard errors, and any two samples with a common letter are not significantly different (*p* > 0.05).

The utilization rates of carbohydrates, carboxylic acids and amino acids by the bacteria of the three PWN isolates were considerably higher than those for the other carbon substrates, but no significant differences were observed in the utilization rates of carbohydrates, carboxylic acids and amines among the bacteria of the three PWN isolates. The bacteria in PdBx had a notably lower utilization rate of aromatic compounds than did the communities from the other two PWN isolates, whereas the amino acids were the C sources most highly utilized by the bacteria of all three *B. xylophilus* isolates. The bacteria in PtBx showed a significantly lower utilization rate of polymers than did the endobactera of the other PWN isolates (Figure 3).

### 2.5. Diversity and Community Structure of Bacteria Associated with PWNs from Different Pine Species after Passage through P. thunbergii

A total of 51,0944 clean reads were obtained from the nematode isolates as a result of high-throughput sequencing. The average length of the assembled reads was 250 bp. Sequences were assigned to OTUs at 97% similarity. The OTUs of bacteria from PmBx, PdBx, PtBx were 1140, 1447 and 1462, respectively. Alpha diversity analyses showed that Shannon-Wiener diversity indices (*H*) of PdBx (6.01), and PtBx (5.55) were higher than that of PmBx (5.27). The species richness index Chao1 values of PtBx (4178) and PdBx (3871) were higher than that of PmBx (3189). These results showed that the diversity and richness of the bacteria in PdBx and PtBx were similar, and higher than the corresponding values for PmBx.

**Figure 4:**
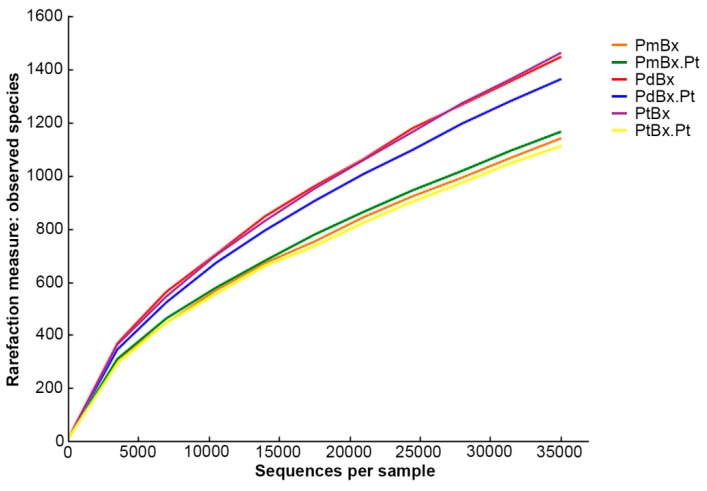
Rarefaction analysis of bacteria associated with different *Bursaphelenchus xylophilus* isolates.

After infection of *P. thunbergii*, the OTUs of PmBx.Pt, PdBx.Pt and PtBx.Pt were 1165, 1363, and 1111, respectively. The Chao1 value and Shannon-Wiener index of PmBx.Pt (3256, 5.42, respectively) increased following passage through *P. thunbergii*, but those of PdBx.Pt (3785, 4.74, respectively) and PtBx.Pt (2847, 5.45, respectively) decreased (Table 2). The rarefaction curves of all samples tended towards saturation (Figure 4). The coverage estimation of each sample was 100, indicating that the OTUs detected were reliable and able to represent the actual bacterial communities.

**Figure 5:**
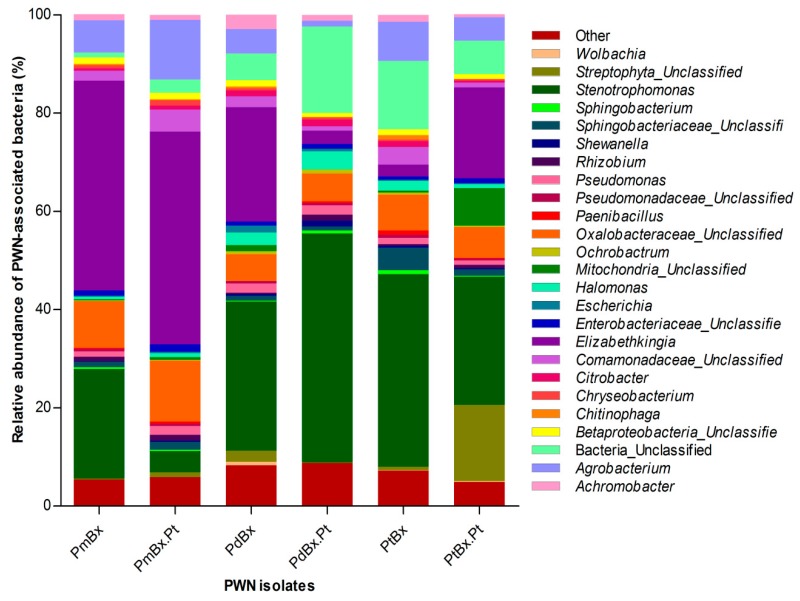
Composition at the genus level of the bacterial communities of *Bursaphelenchus xylophilus* from different PWN isolates.

The most abundant bacteria of PmBx, isolated from *P. massoniana*, were of the genus *Elizabethkingia* (42.7%), while *Stenotrophomonas* was the second (22.3%) most abundant genus, followed by Oxalobacteraceae_Unclassified (9.5%). One percent of bacteria were unclassified. As for the bacteria of PdBx isolated from *P. densiflora*, *Stenotrophomonas* accounted for the greatest proportion (30.3%) followed by *Elizabethkingia* (23.3%), Oxalobacteraceae_Unclassified (5.5%) and *Agrobacterium* (5.0%). In addition, 5.4% of bacteria were unclassified. In PtBx, *Stenotrophomonas* was again the most frequent genus (39.2%), followed by *Agrobacterium* (8.0%), Oxalobacteraceae_Unclassified (7.3%), and Sphingbacteriaceae_Unclassified (4.6%), whereas the remaining 13.4% represented unclassified bacteria. Thus, the predominant bacteria in *B. xylophilus* from the different pine species belonged to *Stenotrophomonas* or *Elizabethkingia* genera (Figure 5).

After re-isolation of the PWNs from *P. thunbergii*, the predominant bacteria were still *Elizabethkingia* in PmBx.Pt (43.3%), *Stenotrophomonas* in PdBx.Pt (46.6%), and *Stenotrophomonas* in PtBx.Pt (26.1%), respectively. The percentage of *Stenotrophomonas* decreased in PmBx.Pt (4.3%) and PtBx.Pt (26.1%), whereas it increased in PdBx.Pt (46.6%). The relative abundance of *Elizabethkingia* decreased in PdBx.Pt (2.7%) and increased in PtBx.Pt (18.5%) but did not change significantly in the relative abundance of PmBx.Pt. The abundance of *Agrobacterium* increased in PmBx.Pt (12.2%) but decreased in PdBx.Pt (1.2%) and in PtBx.Pt (4.8%). Additionally, the relative abundance of Oxalobacteraceae_Unclassified increased in PmBx.Pt (12.4%) but decreased in PtBx.Pt (6.3%), changing slightly in PdBx.Pt (5.7%). Other genera of bacteria in *B. xylophilus*, which each accounted for a small proportion of each bacterial community, did not vary noticeably in relative abundance after being isolated from *P. thunbergii* relative to the situation before inoculation. Additionally, *Paenibacillus* emerged in PdBx.Pt (0.3%), but *Wolbachia* disappeared in PdBx.Pt. following passage through *P. thunbergii*, while bacteria of the genus *Chitinophaga* emerged in PtBx.Pt. There was no change between the communities of bacteria at the genus level associated with PmBx and PmBx.Pt (Figure 5).

### 2.6. Virulence of Different B. xylophilus Isolates Following Inoculation of P. thunbergii

**Figure 6:**
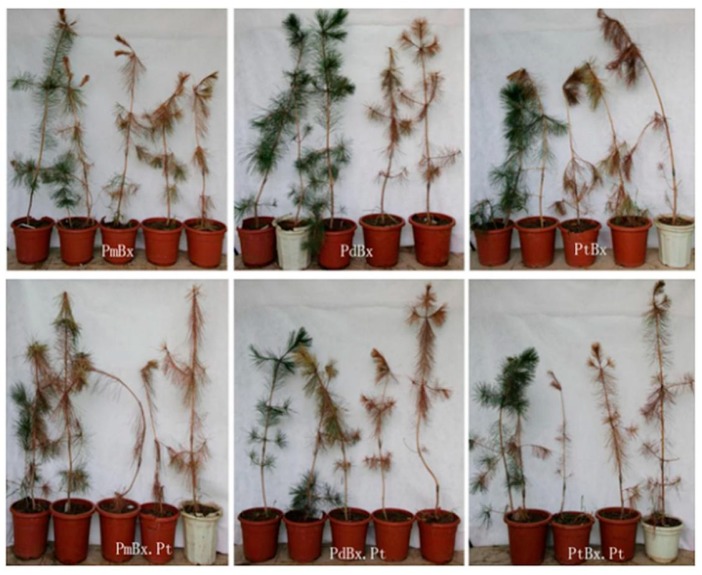
The symptoms of *Pinus thunbergii* after inoculation for 60 d with different *Bursaphelenchus xylophilus* isolates. Each treatment of this experiment has five saplings (The two small saplings in the second pot from the left in PmBx treatment and the leftmost pot in PmBx.Pt treatment was not treated. The two saplings in the leftmost pot in PtBx.Pt treatment was all treated.).

Four-year-old *P. thunbergii* saplings were inoculated with nematode isolates of PmBx, PdBx, PtBx PmBx.Pt, PdBx.Pt, and PtBx.Pt, respectively (Figure 6). Thirty days after inoculation, the infection rate of pine saplings inoculated with PmBx, PdBx and PtBx were 60%, 0% and 20%, respectively. The infection rate of pine saplings inoculated with PmBx.Pt, PdBx.Pt and PtBx.Pt were 80%, 20% and 60%, respectively. The disease severity indexes (DSI) of the pine trees inoculated with PmBx (15), PtBx (0), PtBx (10) were lower than those with PmBx.Pt (70), PdBx.Pt (5), PtBx.Pt (60) (Table 3). Sixty days after inoculation, the infection rates of the pine saplings inoculated with PmBx and PmBx.Pt were both 100%, but the DSIs of those saplings inoculated with PmBx and PmBx.Pt were 65 and 100, respectively, whereas the infection rates of the pine saplings inoculated with PtBx and PtBx.Pt were both 80%, and the DSIs were 75% and 80%, respectively. Also, the infection rate of pine trees inoculated with PdBx was 40%, and DSI was 40, whereas the PdBx.Pt infection rate and DSI of PdBx, Pt were 80% and 75%, respectively (Figure 6, Table 3). These results showed that the virulence of PmBx was the highest, followed by PtBx, with the lowest being PdBx.

## 3. Discussion

It is widely known that PWNs in nature can carry various bacteria. The nematode-associated bacteria in different countries and from different host pine trees are diverse and varied [10]. The species of bacteria carried by PWN may be related to the endophytes of the host plants [20]. In order to understand the relationship between PWN, bacteria and pine species, we collected *B. xylophilus* isolated from *Pinus densiflora*, *P. thunbergii* and *P. massoniana* and sterilized the nematodes’ cuticle. Four species of culturable bacteria were isolated from three PWN isolates. The bacteria of PWN from different pine species varied, with *Stenotrophomonas* and *Pseudomonas* being the main bacterial genera. Wu et al. [18] also reported that *Stenotrophomonas* was the main culturable bacterium of PWN showing differences in virulence.

The Biolog EcoPlate system was used to analyze the metabolic diversity of the bacterial community in *B. xylophilus*. The bacteria in *B. xylophilus* from *P. massoniana* exhibited the highest AWCD, followed by the bacteria in PWN from *P. densiflora*, with the bacteria showing the lowest AWCD being the ones from *P. thunbergii*, although there was no significant difference in the diversity of the bacteria in PWNs isolated from different pine species. Combined with the virulence of PWNs isolated from different pine species, these results showed that the most virulent PWN carried bacteria with an ability to utilize a wider range of carbon substrates. Wu et al. [18] also found that carbon metabolism of the bacteria in *B. xylophilus* might be related to the virulence of *B. xylophilus.* In their study, the bacteria in highly virulent *B. xylophilus* had a relatively high utilization rate of carbohydrate and carboxylic acids, while bacteria in poorly virulent *B. xylophilus* made better use of amino acids. In the current study, there were no significant differences among the bacteria in the three PWN isolates in terms of the utilization of carbohydrates, carboxylic acids and amines. But the ability of bacteria in PWN from *P. densiflora* to utilize aromatic compounds was significantly lower than that in the other two isolates. Phenolic acids, as types of aromatic compounds, including benzoic acid and salicylic acid, are closely associated with host plant resistance [21]. The activities of defense enzymes secreted by different pine tree species have been shown to be different [22,23,24]. Those bacteria, which could better utilize phenolic acids, might contribute to diminishing the host plant’s defensive effects and help the nematodes to survive within the tree.

In comparison with traditional culture methods, high-throughput sequencing can be used to analyze unculturable bacteria. The results showed that there were differences in diversity and relative abundance of bacteria associated with PWN from different pine species. Xiang et al. [19] found that the diversity and relative abundance of bacteria associated with PWN were related to the virulence of the *B. xylophilus* isolate. Testing the virulence of different PWN isolates showed that PmBx was the most virulent, followed by PtBx and PdBx. In PmBx, the most abundant genus of bacteria was *Elizabethkingia*. *Elizabethkingia* was also the dominant midgut bacterial genus in mosquito that could tolerate high pH and/or oxidative stress [25,26,27]. In the earliest stages of PWD, pines accumulate reactive oxygen species (ROS) as part of a defense response [28]. The PWN is able to tolerate ROS attack by producing several antioxidant enzymes [29,30]. In addition, some PWN-associated bacteria could increase PWN survival under strong oxidative stress [31]. Therefore, it was inferred that *Elizabethkingia* could resist high oxidative stress in order to help *B. xylophilus* survival. In PtBx and PdBx, the most abundant genus of bacteria was *Stenotrophomonas*. *Stenotrophomonas* can degrade the insect-repellent terpene α-pinene from pines and regulate the expression of some pathogenesis-related genes in *B. xylophilus* to affect the development and virulence of *B. xylophilus* [10,32]. These most abundant bacteria in *B. xylophilus* might have a supporting effect on the virulence of *B. xylophilus*.

Mamiya et al. [33] found that a large number of bacteria would gather around PWN after pine trees were inoculated with nematodes. In our study, following infection of *P*. thunbergii and subsequent re-isolation, bacterial diversity increased in PmBx.Pt, but decreased in both PtBx.Pt and PdBx.Pt. The relative abundances of some of the major PWN-associated bacteria, such as *Stenotrophomonas*, Oxalobacteraceae_Unclassified, *Elizabethkingia* and *Agrobacterium*, changed considerably. In addition, some bacterial genera were unique to the PWN isolate which had infected *P. thunbergii*, such as *Bacillus*, *Paenibacillus* and *Chitinophaga*, suggesting that these bacteria were obtained by the nematode in the process of the PWN infection of *P. thunbergii*. The virulence of PWN isolates from different host pine species all increased after passage through *P. thunbergii*, suggesting that infecting pine may help PWN improve its parasitism and virulence. The level of PWN virulence might be related to the species of and carbon utilization by bacteria associated with PWN [18]. PWN-associated bacteria could secrete cellulases to degrade pine cell walls for better PWN colonization [13,14] and could improve nematode survival by degrading and/or detoxifying the pine defense compounds [31,32,34]. The changes in the community composition of the bacteria associated with PWN after inoculating *P. thunbergii* might help to adjust the PWN’s parasitic adaptability. However, whether or not there is a selection mechanism between *B. xylophilus* and PWN-associated bacteria remains to be the subject for further study.

## 4. Materials and Methods

### 4.1. Collection and Surface Sterilization of Nematodes

Symptomatic adult pine trees were selected in locations known to be affected by PWD. *P. massoniana* trees used in this study were from the Tianmuhu forest plantation in Nanjing, Jiangsu, China. The nematodes (PmBx) were isolated from the logs by the Baermann funnel method. *P. densiflora* and *P. thunbergii* trees were from Jurong forest plantation in Zhenjiang. Two isolates of *B. xylophilus* (PdBx from *P. densiflora* and PtBx from *P. thunbergii*) were isolated from their logs. The three isolates of PWN were preserved at the Jiangsu Key Laboratory for Prevention and Management of Invasive Species.

The nematodes were grown on *Botrytis cinerea* cultured on PDA plates for 4–5 d at 25 °C. They were extracted overnight using the Baermann funnel technique and washed three times with sterilized distilled water (DW). Nematodes were surface sterilized with 1% mercuric chloride for 30 min followed by rinsing with DW and soaked in a mixture of 2% spectinomycin and 2% gentamicin for 30 min, followed by rinsing with DW. Afterwards, the nematodes were transferred to nutrient agar (NA) plates at 28 °C for 48 h to check for the presence of any bacterial colonies.

### 4.2. Isolation and Identification of Culturable Bacteria from Nematodes

Culturable bacteria were isolated from surface-sterilized *B. xylophilus* using a previously reported method [35]. The bacterial strains were maintained in nutrient broth (NB) liquid medium at 28 °C for 18 h. Total genomic DNA was extracted from the bacteria of *B. xylophilus* by EasyPure Genomic DNA Kit (TransGen Biotech, China). The 16S rDNA was amplified using primer pairs of 27F (5’-AGAGTTTGATCCTGGCTCAG-3’) and 1492R (5’-TACCTTGTTACGACTT-3’). The PCR reaction was performed according to the method described by Yuan et al. [35], after which the PCR products were sequenced by Jinsite Ltd, Nanjing, China. The similarity of the 16S rDNA partial gene sequence was compared with the existing sequences available in the NCBI GenBank database, using a BLAST search. The sequence alignment was carried out using ClustalW and the phylogenetic tree was constructed by MEGA 7.0 using the Maximum-Likelihood method based on the Kimura 2-parameter (K2P) model [36]. Bootstrap analysis was performed with 1000 replicates.

### 4.3. Biolog EcoPlate Measurement of Bacterial Community of Nematodes

The functional diversity of bacterial community of surface sterilized nematode was measured with Biolog EcoPlate microplates (Biolog, Hayward, CA, USA). Each plate had 32 wells including 31 carbon © sources and one well of water, with three parallel repetitions in a 96-well microplate. A sample of 50,000 cuticle-sterile *B. xylophilus* were ground with an aseptic motorized homogenizer and silica and the final volume was adjusted to 15 mL with DW. An aliquot (100 μL) of suspension was pipetted into each well of the microplate, which was incubated at 25 °C in the dark under humid conditions. Optical absorbance was measured at 590 nm with an Emax precision microplate reader every 24 h for 168 h. The values of the absorbance readings for individual wells of the Biolog Eco-plates were presented as average well color development (AWCD) according to Garland and Mills [37]. The AWCD, reflecting the total metabolic activities of the microbial community on the individual C substrates, was calculated using the following formula: AWCD_590nm_ = ∑(ni)/31, where ni is the metabolic activity on each substrate. The Shannon-Wiener index (*H*) and Evenness index (*E*) were calculated as:

H = −∑(Pi × lnPi)

where Pi = C590 − 750/∑(C590 − 750);.

E = H/lnS,

where P_i_ is the ratio of ni to the sum of activities on all substrates, and S is the species richness of the community [38,39].

### 4.4. Inoculation of P. thunbergii with B. xylophilus

The nematodes cultured on *Botrytis cinerea* for six days at 25 °C were harvested using a Baermann funnel. The nematodes were sterilized in 0.2% streptomycin sulfate for 30 min and washed three times with DW.

The stems of four-year-old *P. thunbergii* saplings were cut at 10–15 cm above the base of the tree with a sterilized scalpel. A piece of sterile absorbent cotton wool was placed on each wound, and 0.2 mL of the inoculum suspension liquid (containing 8000 nematodes in DW) was pipetted onto the cotton. The wounds were covered by parafilm. Inoculation with 0.2 mL DW was used as the negative control. Each treatment had five replicates and saplings were placed in greenhouse at 25 °C.

Sixty days after inoculation, the pine saplings were cut into about 1.5 cm long wood sections. The nematodes were isolated using the Baermann funnel method. *B. xylophilus* PmBx.Pt, PdBx.Pt and PtBx.Pt were isolated from *P. thunbergii* inoculated with PmBx, PdBx and PtBx, respectively.

### 4.5. High-Throughput Sequencing of Bacterial Community of Nematodes

About 5,0000 cuticle-sterilized nematodes from each isolate were ground in a sterile mortar with liquid nitrogen. The ground nematodes were transferred to a 1.5 mL centrifuge tube and the total genomic DNA was extracted use E.Z.N.A. Mollusc DNA Kit (Omega, Norcross, GA, USA) following the manufacturer’s instructions.

PCR primers 515f/806r targeting the bacterial 16S rDNA V4 region were used in PCR amplification [40]. To distinguish among the different samples, a Barcoded-tag with six nucleotide bases was randomly added upstream of the universal primer. The primers which had Barcoded-tag sequences added were Barcoded-tag fusion primers (BFP). After quantification and quality control, 16S rDNA PCR products were paired-end sequenced with the Illumina MiSeq platform (Illumina, San Diego, CA, USA).

The lengths of the short reads were extended by identifying the overlap between paired-end reads using the FLASH software [41]. The reads were filtered by QIIME quality filters [42]. Sequencing reads were sorted according to barcode sequences and the sample sources. The number of reads of each sample was counted. Sequences were assigned to operational taxonomic units (OTUs) at 97% similarity by using UPARSE software [43]. A representative sequence for each OTU was picked and taxonomic data were assigned to each representative sequence by the Ribosomal Database Project (RDP) classifier [44]. To compute the alpha diversity, species richness (Chao1), species coverage (Coverage, C) and species diversity (Shannon-Wiener Index, H) were calculated. Python script alpha_rarefaction.py from QIIME-1.7.0 was used for diversity index analysis with the default parameter setting. The richness index Chao1 was used to estimate the number of OTUs in the bacterial communities. The Shannon-Wiener Index was used to estimate the diversity of the OTUs. Community structure analyses were based on the genus taxonomic level.

### 4.6. Virulence Test of the Nematode Isolates

The nematode isolates PmBx, PdBx, PtBx, PmBx.Pt, PdBx.Pt and PtBx.Pt were used to inoculate four-year-old *Pinus thunbergii* saplings with the same inoculation method as in Section 4.4. Inoculation with 0.2 mL DW was used as the negative control. Each treatment had five replicates. The disease development was recorded at 2 d intervals. The infection rate is the prevalence of the disease. The disease severity of *P. thunbergii* infection was classified into five levels: 0, the trees healthy with green needles and growing well; 1, a few needles turning brown; 2, half of the needles turning brown and the terminal shoots of the trees bending; 3, most of the needles turning brown and dead and the terminal shoots of trees drooping; 4, all of the needles turning brown and the whole tree wilting. The infection rates and the disease severity index were calculated according to He et al. [16].

$$\mathrm{Infection}\;\mathrm{rate}\;\left(\%\right)\;=\;\frac{\sum \mathrm{number}\;\mathrm{of}\;\mathrm{infected}\;\mathrm{plants}\;\mathrm{with}\;\mathrm{symptoms}}{\mathrm{Total}\;\mathrm{number}\;\mathrm{of}\;\mathrm{plants}}\;\times \;100$$

$$\mathrm{Disease}\;\mathrm{severity}\;\mathrm{index}\;\left(\mathrm{DSI}\right)\;=\;\frac{\sum \mathrm{number}\;\mathrm{of}\;\mathrm{diseased}\;\mathrm{plants}\;\times \;\mathrm{symptom}\;\mathrm{stage}}{\mathrm{Total}\;\mathrm{number}\;\mathrm{of}\;\mathrm{plants}\;\times \;\mathrm{highest}\;\mathrm{symptom}\;\mathrm{stage}}\;\times \;100$$

### 4.7. Statistical Analysis

All data were presented as the mean ± standard deviation (mean ± S.D.). All parameters were calculated using Microsoft Excel. The analysis of variance (ANOVA) plus Tukey test was performed using SPSS Statistics 24.0 software (IBM China Company Ltd, Beijing, China). The threshold level of significance was *p* ≤ 0.05.

The datasets generated and analyzed during the current study are available in the GenBank (KJ748599- KJ748604; available at: https://www.ncbi.nlm.nih.gov/nuccore).

## Figures and Tables

**Table 1:** Ecological diversity of endo-bacteria communities of *Bursaphelenchus xylophilus* from different pine host species.

*B. xylophilus*	Diversity Index	
	Shannon-Wiener (*H*)	Evenness (*E*)
PmBx	2.760 ± 0.334 ^a^	0.086 ± 0.011 ^b^
PdBx	2.965 ± 0.082 ^a^	0.114 ± 0.003 ^a^
PtBx	2.506 ± 0.043 ^a^	0.104 ± 0.013 ^ab^

Data in the table was the mean ± standard deviation (partial); *n* = 3; Any two samples with a common letter are not significantly different, *p* > 0.05, following ANOVA and the Tukey test.

**Table 2:** Diversity index of bacteria associated with different *Bursaphelenchus xylophilus* isolates.

*B. xylophilus*	OTU (97%)	Coverage (*C*) %	Chao1 (97%)	Shannon-Wiener(*H*) (97%)
PmBx	1140	100	3189	5.27
PmBx.Pt	1165	100	3256	5.42
PdBx	1447	100	3871	6.01
PdBx.Pt	1363	100	3785	4.74
PtBx	1462	100	4178	5.55
PtBx.Pt	1111	100	2847	5.45

**Table 3:** The symptoms at 30 d and 60 d of *Pinus thunbergii* inoculated with different *Bursaphelenchus xylophilus* isolates.

Nematode Strains	Infection rates (%)		DSI	
	30 d	60 d	30 d	60 d
PmBx	60	100	15	65
PmBx.Pt	80	100	70	100
PdBx	0	40	0	40
PdBx.Pt	20	80	5	75
PtBx	20	80	10	75
PtBx.Pt	60	80	60	80

## Abbreviations

PWN	Pine wood nematode
PWD	Pine wilt disease
rDNA	Ribosomal deoxyribonucleic acid
NA	Nutrient agar
NB	Nutrient broth
PCR	Polymerase chain reaction
NCBI	National center for biotechnology information
BLAST	Basic local alignment search tool
MEGA	Molecular evolutionary genetic analysis
K2P	Kimura 2-parameter
AWCD	Average well color development
DNA	Deoxyribonucleic acid
BFP	Barcoded-tag fusion primer
OTU	Operational taxonomic unit
RDP	Ribosomal database project
DSI	Disease severity index
ROS	Reactive oxygen species
